# Self-Expanding Metal Stenting for Palliation of Patients with Malignant Colonic Obstruction: Effectiveness and Efficacy on 255 Patients with 12-Month's Follow-up

**DOI:** 10.1155/2012/296347

**Published:** 2012-06-11

**Authors:** Søren Meisner, Ferran González-Huix, Jo G. Vandervoort, Alessandro Repici, Dimitrios Xinopoulos, Karl E. Grund, Paul Goldberg, The WallFlex Colonic Registry Group

**Affiliations:** ^1^Endoscopy Unit, Bispebjerg Hospital, Bispebjerg Bakke 23, Entrance 7B, 2400 Copenhagen NV, Denmark; ^2^Unidad de Endoscopia, Servicio de Aparato Digestivo, Hospital Doctor Josep Trueta, Carretera Franca s/n, Catalunya, 17007 Girona, Spain; ^3^Gastro-enterologie, Onze Lieve Vrouw Ziekenhuis, Moorselbaan 164, 9300 Aalst, Belgium; ^4^Department of Digestive Endoscopy, Istituto Clinico Humanitas, Via Manzoni 56, 20089 Rozzano, Italy; ^5^Gastroenterology Unit, Saint Savas Hospital, 171 Alexadras Avenue, 11522 Athens, Greece; ^6^Department of Surgical Endoscopy, University Hospital Tuebingen, Geissweg 3, 72076 Tuebingen, Germany; ^7^Department-Colorectal Surgery, Groote Schuur Hospital, Private Bag, Observatory, Cape Town 7937, South Africa; ^8^WallFlex Enteral Colonic Stent for Relieving Malignant Colorectal Obstruction, Boston Scientific, Natick, MA 01760, USA

## Abstract

*Background*. Self-expanding metal stents can alleviate malignant colonic obstruction in incurable patients and avoid palliative stoma surgery. *Objective*. Evaluate stent effectiveness and safety on palliation of patients with malignant colorectal strictures. *Design*. Two prospective, one Spanish and one global, multicenter studies. *Settings*. 39 centers (22 academic, 17 community hospitals) from 13 countries. *Patients*. A total of 257 patients were enrolled, and 255 patients were treated with a WallFlex uncovered enteral colonic stent. Follow-up was up to 12 months or until death or retreatment. *Interventions(s)*. Self-expanding metal stent placement. *Main Outcome Measures*. Procedural success, clinical success, and safety. *Results*. Procedural success was 98.4% (251). Clinical success rates were 87.8% at 30 days, 89.7% at 3 months, 92.8% at 6 months, and 96% at 12 months. Overall perforation rate was 5.1%. Overall migration rate was 5.5%. Overall death rate during follow-up was 48.6% (124), with 67.7% of deaths related to the patient's colorectal cancer, unrelated in 32.3%. Only 2 deaths were related to the stent or procedure. *Limitations*. No control group. *Conclusions*. The primary palliative option for patients with malignant colonic obstruction should be self-expanding metal stent placement due to high rates of technical success and efficacy in symptom palliation and few complications.

## 1. Introduction

Colorectal cancer is the second most prevalent cancer in the world with incidence of 1 million new cases per year and mortality of about 529,000 deaths [1].

Obstruction has been reported in 7–29% of patients with colorectal cancer [2]. Patients with malignant large-bowel obstruction tend to have advanced disease and be poor surgical candidates.

Surgical colostomy is effective, but mortality can be high. In addition, a colostomy diminishes quality of life, and its management poses difficulties for elderly and frail patients [3, 4].

The patient presents either with acute “total” obstruction or subacute bowel obstruction. Severity of symptoms may vary. Conventional therapies for relieving malignant colorectal obstruction include surgical resection (potentially curative) or palliative colostomy. Resection is ideally carried out as a single-stage procedure, with anastomosis to restore bowel continuity, but multistage procedures may also be undertaken, with resection and stoma formation in one procedure, followed by restoration of continuity in another procedure. However, a significant proportion of patients, up to 50%, receiving a staged procedure never undergo reversal of the colostomy [5]. In the emergency setting, surgery carries a high-mortality (15–20%) and high-morbidity (45–50%) risk with increased prevalence of intensive care stay, infections, and complications related to stomas [4].

Endoscopic placement of SEMS to relieve colonic obstruction has been introduced more than 10 years ago [6]. Colonic SEMS may be used for palliation to eliminate the need for stomas in incurable patients or to “bridge” patients to elective single-stage surgery, most often without a stoma, thereby significantly reducing the mortality and morbidity [7–9].

Although effectiveness of the use of colonic SEMS in a palliative or bridge to surgery indication is fairly broadly recognized, some have questioned the safety of colonic stenting, particularly as it pertains to colonic perforation [10]. This paper reports on the largest prospective series of treatment with colonic SEMS per local standard of practice. The focus of analysis is on broadness of applicability of the method and patient safety.

## 2. Materials and Methods

### 2.1. Study Design

Two prospective, one Spanish and one global, multi-center studies on the safety and effectiveness of the WallFlex Enteral Colonic Stent (Boston Scientific, Natick, MA, USA) were conducted using two identically structured registries, the WallFlex-eR Colonic International Registry and the WallFlex-eR Colonic Spanish Registry. Data was collected over the course of 2 years and 9 months (international registry) and 1 year and 11 months (Spanish registry). Thirty-nine (39) centers (22 academic, 17 community hospitals) from 13 countries enrolled patients with colorectal strictures secondary to malignant disease who required stent treatment either for a palliative (PAL) or bridge-to-surgery (BTS) indication. Each patient was treated according to the product directions for use by one of the 35 (89.7%) gastroenterologists and 5 (12.8%) surgeons in the study.

This study was conducted as a registry where data was collected for procedures performed per standard of practice. Institutional review board (IRB) approval of the protocol was obtained as required. Per guidance from their local IRB departments, some centers did not require IRB approval citing the registry nature of the studies, the use of a non-investigational (approved) device, and treatment that is standard of care for each patient included in the registries. Documentation of patient consent to the procedure as well as to the participation in the study or IRB waiver of the need of such consenting to study participation was obtained.

Patient assessments were performed per each center's usual medical practices and according to the registry protocol. Data was collected at the time of stent placement and included patient demographics, symptoms of colonic obstruction, ability to pass stool, treatments prior to stenting, tumor characteristics such as origin and location, stent placement, and procedure details. Palliative patients were followed for up to 12 months, namely at 30 days, 3 months, 6 months, and 12 months, or until death or retreatment, whichever occurred first. Data collected during those visits included symptoms of obstruction, stool passage, and retreatment details. Complications and adverse events were collected and documented for each patient as they occurred. Data was prospectively added to the registries after each visit.

### 2.2. Patient Population

Patients who met the inclusion criteria of presenting with acute or symptomatic colonic obstruction secondary to malignant neoplasms and requiring either palliative or bridge-to-surgery treatment with a stent were enrolled.

Exclusion criteria consisted of the following: placement of a previous colonic stent, enteral ischemia, suspected or impending perforation, intra-abdominal abscess/perforation, contraindication to endoscopic treatment, and any use of the stent other than those specifically outlined under indications of use.

At the time of enrollment, patients were determined to be either palliative (PAL) or bridge to surgery (BTS) based on the malignant disease stage, age, surgical risk, and in some cases due to patient's choice. Data from the PAL cohort only is presented in this paper.

### 2.3. Device and Stenting Procedure

SEMSs were placed by the aid of fluoroscopy and under direct visualization with an endoscope. Conscious sedation was employed during the majority of procedures in 80% (204) cases, and general anesthesia was used on 12 patients. A 0.035-inch guidewire was passed across the site of the stricture. The size of the stricture was estimated, and the length and number of stents needed to cross the stricture were determined. In a small group of patients (9), the stricture was dilated prior to stent placement using either a balloon or bougie dilator. The WallFlex Enteral Colonic Stent delivery system was then passed over the guidewire, through the endoscope working channel and to the site of the stricture until the postdeployment marker band was at the proximal end of the stricture. The exterior tube marker band was used to position the stent at the distal end of the stricture. The stent was then deployed.

### 2.4. Outcomes Measures

#### 2.4.1. Procedural Success

Procedural success was evaluated as a measure of successful endoscopic placement of the stent in correct position.

#### 2.4.2. Clinical Success

Effectiveness of the stent was measured by its clinical success on the patient, defined as providing adequate passage of stool (excellent, good, or fair) from stent placement until death or 12 months without the occurrence of any device or procedure-related complications.

#### 2.4.3. Safety Profiles

Major complications such as perforations and migrations were reported as they occurred and were grouped in separate categories based on timing: up to 6 hours after procedure and at each follow up point (30 days, 3 months, 6 months and 12 months).

### 2.5. Statistical Analyses

Summary statistics were computed for either the *enrolled* population or the *treated *population, depending on the measure being summarized. For categorical measures at each visit, summary statistics consist of frequency and percent of responses in each category. Unless otherwise noted, the denominator of a percentage is the number of subjects with nonmissing values, based on available follow-up data (*evaluable* patients). For continuous measures at each visit, summary statistics include sample size, mean, median, standard deviation, minimum, and maximum. The sample size is the number of nonmissing values, based on evaluable patients, unless noted otherwise.

## 3. Results

Only results from the registries PAL cohort (255 patients stented) are presented in this paper. Data from the registries have been presented in two previous publications, one presenting 30-day safety and efficacy results including all patients (447 patients) [11] and one presenting outcome results of the BTS cohort (182 patients) [12].

### 3.1. Baseline Characteristics

In total, 257 patients, 141 from the WallFlex-eR Colonic International Registry and 116 from the Spanish Registry, were enrolled with the intention of a palliative treatment with a colonic stent. WallFlex colonic stents were placed in 255 of the enrolled patients.

The cohort consisted of 155 (60.3%) male patients with patient mean age of 73.5 ± 13.0 and average BMI of 25.1 ± 4.2. Reported ASA scores were I in 17 (6.6%) cases, II in 99 (38.5%), III in 96 (37.4%), IV in 28 (10.9%) and V in 1 (0.4%) cases. Symptoms of colonic obstruction were presented as follows: nausea in 93 (36.9%) patients, vomiting in 67 (26.7%), constipation in 192 (77.1%), diarrhea in 66 (26.4%), abdominal pain/cramps in 190 (75.4%), and bloating in 144 (57.1%) patients. At baseline, previous treatments for colo-rectal cancer were recorded in 38 (15%) of patients, with the majority having undergone chemotherapy (24), surgical resection (16), and/or radiation (3). Previous lower abdominal and/or pelvic surgery was reported in 57 (22.4%) patients, and 27 (10.6%) patients had history of chemotherapy and/or radiation for other malignant noncolorectal disease.

Tumor etiology was described as intrinsic in 226 (89.3%) and extrinsic in 27 (10.7%) of cases. The colo-rectal tumor was mostly found in the left-sided colon (72.9%) which included rectosigmoid junction, sigmoid colon, descending colon, and splenic flexure; rectal tumors were seen in 19.2% of the cases and 10.2% in the proximal colon which included transverse colon, hepatic flexure, and ascending colon. The most common dissemination of disease was reported as liver metastasis in 138 (53.7%) patients, while 97 (37.7%) patients had multiple metastases. Only 64 (24.9%) patients had local colo-rectal cancer without proven metastasis, and decision on palliative stent treatment was made in poor operative candidates with severe comorbid medical illnesses.

### 3.2. Procedure Details

Twelve of the 255 treated patients required two stents, and one patient received three stents. The most commonly used stent size was of 9 cm length and 25/30 mm body/flare diameter.

Prestent placement dilation was done in 9 (3.5%) patients using either balloon or bougie. After stent deployment, the scope was passed through the stent in 43 (16.9%) patients.

### 3.3. Follow-Up Visits

Of the 255 stented patients, 206 had a 30-day follow-up visit, 126 were still being followed at 3 months, 86 at 6 months, and 36 patients reached the 12-month follow-up point. Obstruction symptoms and passage of stool of patients with evaluable data at each follow-up interval are reported in Table 1.

### 3.4. Procedural Success

Procedural success was achieved in 98.4% (251) of cases, of which 91.4% (233) were regarded as having stents in optimal position.

### 3.5. Clinical Success

Clinical success, as a measure of bowel function and lack of stent-related complications, was assessed on 206, 126, 86, and 36 patients who had follow up visits at 30 days, 3 months, 6 months, and 12 months, respectively. However, some of those patients were not evaluable for clinical success due to either missing data on bowel function or due to being lost to follow-up or death, thus making the clinical success evaluable cohorts equal to 196 at 30 days, 116 at 3 months, 69 at 6 months, and 25 at 12 months.

The overall clinical success rate ranged from 87.8% to 96% depending on the follow-up period (87.8% at 30 days, 89.7% at 3 months, 92.8% at 6 months, and 96% at 12 months). Reasons for clinical failure are outlined in Table 2.

### 3.6. Safety

The overall perforation rate was 5.1% (13/255).  Table 3 lists the adverse events at each follow-up point. Perforation rates were 1.2% at procedure, 3.3% at 30 days, 1.4% at 3 months, 0% at 6 months, and 2.5% at 12 months. There was 1 stent migration (0.4%) at the time of procedure, 5 (2.3%) at 30 days, 4 (2.9%) at 3 months, 4 (4.0%) at 6 months, and no migrations at 12 months. The overall migration rate was 5.5% (14/255). The main cause of reobstruction was tumor in—or overgrowth (17%).

#### 3.6.1. Mortality

One hundred and twenty-four (124) of the 255 palliative patients died before the end of the 12-month follow-up period. Reasons for death were attributed to the patients' colo-rectal cancer in 84 (67.7%) cases and not related to colo-rectal cancer in 40 (32.4%) patients. Only 2 deaths were related to the stent or procedure after a perforation treated by surgery that occurred in one patient at day 24 and in another patient at day 34 after stenting.

## 4. Discussion

SEMSs for palliation of malignant colo-rectal obstruction are placed in patients with extensive metastatic disease and in poor operative candidates with severe comorbid medical illnesses.

**Figure 1:**
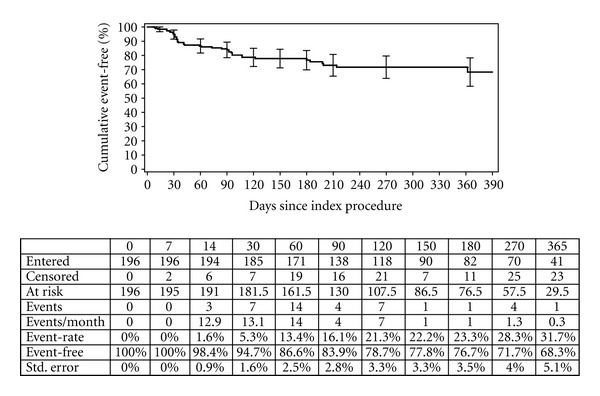
The Kaplan-Meier analysis of time to clinical failure on treated patients.

Numerous series on the use of SEMSs have been published, and many have been included in several reviews [7–9]. Our results on the effectiveness and efficacy of the WallFlex colonic stent are similar to previously reported SEMS outcome results. Adverse events after stent placement are common, from the serious perforation to minor clinical problems with impaired bowel function. To illustrate the benefits of palliation with stents, we performed a Kaplan-Meier analysis of time to clinical failure on treated patients shown in Figure 1. After 6 months more than 75% of treated patients were event-free.

Regardless of the lack a of published large randomized controlled trials on SEMSs, our data, together with previously reported data, suggests that SEMSs are the option of choice in the initial management of patients with obstructing colon cancer and nonresectable metastases.

A recent publication from Nagula et al. [13] describes a prospective observational cohort study of patients with advanced malignancies undergoing either colon stent insertion or surgical diversion for malignant large-bowel obstruction. The study was initially designed as a randomized trial of colonic stent insertion and surgical palliation with an observational arm for patients declining randomization. Because of patient reluctance to being randomized and the referring physician's preference for either surgery or stent placement, no patients were enrolled in the randomized trial. The data was instead used to assess quality of life (QoL) and longitudinal symptom control in patients after colon stent placement or surgical diversion. The study showed that neither stent placement nor surgery was associated with improvement in overall QoL, but stent placement was associated with improved QoL related to gastrointestinal function.

The decision for palliative care of patients with extensive metastatic disease and those who are poor operative candidates should be individualized to each patient. The high morbidity and mortality associated with emergency surgery should be avoided. The patient's present and future need for chemotherapy also favors stent placement as it ensures that immediate postprocedure chemotherapy will not be delayed by a surgical procedure with the risk of even additional delay due to complications.

Karoui et al. [14] analyzed the impact of stent placement versus surgery with special reference to time to chemotherapy administration. Patients who were treated with an SEMS had shorter hospital stays than patients who had surgery (median, 8.0 versus 13.5 days). In addition, the incidence of stoma creation was lower compared to patients treated with surgery (6% versus 37%). The median time to chemotherapy administration was shorter after SEMS insertion than after surgery (14.0 versus 28.5 days). There was no difference in survival between the 2 groups. The authors reported a tumor perforation requiring emergency surgery in two patients (6%) treated with a SEMS and undergoing chemotherapy and pointed out that the risk of tumor perforation while receiving chemotherapy requires further attention. Later publications also highlight this, especially if drugs like Avastin (bevacizumab) where the antiangiogenic effect may weaken the bowel wall and predispose the bowel to perforation by SEMS pressure [15].

Faster and less complicated recovery after SEMS treatment is also reported in a newly published paper by Vemulapalli et al. [16]. The authors retrospectively reviewed and compared data from patients with obstructing colon cancer who either underwent insertion of a SEMS (*n* = 53) or had surgery (*n* = 70) from 2002 to 2008. Patients in the SEMS group had a significantly shorter median hospital stay (2 days) compared to the surgery group (8 days). In addition; patients with SEMS had significantly fewer acute complications compared to the surgery group (8 versus 30%). Furthermore, the hospital mortality for the SEMS group was 0% compared to 8.5% in patients who underwent surgical decompression. There was no difference in survival rates between the two groups.

## 5. Conclusions

Our data on effectiveness and efficacy on the use of self-expanding metal stents for palliation of patients with malignant colonic obstruction strongly support that stent placement should be used as the primary palliative option, due to the high technical success rate, low complication rate and high efficacy in symptom palliation.

## Figures and Tables

**Table 1:** Patient characteristics.

Measure	Baseline	30-day follow-up	3-month follow-up	6-month follow-up	12-month follow-up
*Symptoms of obstruction*					
Nausea	36.9% (93/252)	7.9% (15/191)	4.8% (6/125)	3.5% (3/85)	16.7% (6/36)
Vomiting	26.7% (67/251)	4.7% (9/192)	1.6% (2/125)	3.6% (3/84)	5.6% (2/36)
Constipation	77.1% (192/249)	17.2% (33/192)	13.6% (17/125)	20.0% (17/85)	28.6% (10/35)
Diarrhea	26.4% (66/250)	17.2% (33/192)	11.2% (14/125)	10.7% (9/84)	11.4% (4/35)
Abdominal pain/cramps	75.4% (190/252)	23.0% (44/191)	22.6% (28/124)	15.7% (13/83)	19.4% (7/36)
Bloating	57.1% (144/252)	12.6% (24/191)	4.8% (6/125)	7.1% (6/84)	11.4% (4/35)

*Passage of stool*					
Good	5.1% (13/255)	77.7% (160/206)	84.1% (106/126)	83.7% (72/86)	75.0% (27/36)
Fair	9.4% (24/255)	11.2% (23/206)	11.1% (14/126)	11.6% (10/86)	19.4% (7/36)
Poor	83.9% (214/255)	7.8% (16/206)	3.2% (4/126)	1.2% (1/86)	2.8% (1/36)
Unknown	1.2% (3/255)	3.4% (7/206)	1.6% (2/126)	3.5% (3/86)	2.8% (1/36)
Missing	0.4% (1/255)	0.0% (0/255)	0.0% (0/255)	0.0% (0/255)	0.0% (0/255)
Lost to follow-up/death	0.0% (0/255)	19.2% (49/255)	50.6% (129/255)	66.3% (169/255)	85.9% (219/255)

**Table 2:** Factors contributing to clinical failure.

Reason for Clinical Failure	Follow-up interval			
	30-day *N* = 24	3-month *N* = 12	6-month *N* = 5	12-month *N* = 1
Poor ability to pass stool	9	3	1	—
Complication/AE since stent placement	10	9	4	1
Poor stool and a complication/AE since stent placement	5	—	—	—

**Table 3:** Procedural and cumulative safety data.

Adverse event	Procedure (*n* = 255)	Cumulative (*n* = 94)
Perforation	1.2% (3/255)	13.8% (13/94)
Stent migration	0.4% (1/255)	12.8% (12/94)
Reobstruction due to		
Ingrowth	—	10.6% (10/94)
Overgrowth	—	6.4% (6/94)
Faecal impaction	—	8.5% (8/94)
Mucosal/bowel impaction into stent oral	—	3.2% (3/94)
Mucosal/bowel impaction into stent anal	—	1.1% (1/94)
Stent patent but second colonic obstruction	—	2.1% (2/94)
Bleeding	0.4% (1/255)	4.3% (4/94)
Pain	1.2% (3/255)	4.3% (4/94)
Persistent obstruction	0.4% (1/255)	1.1% (1/94)
Other	—	21.3% (20/94)
Total AEs	3.5% (9/255)	74.5% (70/94)
